# A Stream Function Smoothing Method for the Design of MRI Gradient Coils on Non-Developable Surfaces

**DOI:** 10.3390/s23187912

**Published:** 2023-09-15

**Authors:** Bohan Yang, Hao Ren, Tongxing Zuo, Zhenyu Liu

**Affiliations:** 1 Changchun Institute of Optics, Fine Mechanics and Physics, Chinese Academy of Sciences, Changchun 130033, China; yangbohan19@mails.ucas.ac.cn (B.Y.);; 2 China School of Optoelectronics, University of Chinese Academy of Sciences, Beijing 100049, China; 3 Department of Mechanical Systems Engineering, Graduate School of Engineering, Nagoya University, Nagoya 464-8601, Japan

**Keywords:** MRI gradient coils, non-developable surface, stream function smoothing method, implicit function diffusion equation

## Abstract

Insert gradient coils with similar imaging body shapes typically have smaller dimensions and higher spatial efficiency. This often allows the gradient coils the achievement of stronger and faster gradient fields. Thus, improving existing methods to make them applicable to the design of MRI gradient coils on complex surfaces has also become a challenge. This article proposes an algorithm that smooths the implicitly expressed stream function based on the intrinsic surface Laplace–Beltrami operator. This algorithm can be used to simplify the design procedure of MRI gradient coils on non-developable surfaces. The following steps are performed by the proposed algorithm: an initial design of the stream function configuration, extraction of the surface mesh, discretization of the surface smoothing operator, and a smoothing of the contour lines. To evaluate the quality of the smoothed streamline configuration, several technical parameter metrics—including magnetic field accuracy, coil power consumption, theoretical minimum wire spacing, and the maximum curvature of the contour lines—were evaluated. The proposed method was successfully validated in a design gradient coil on both developable and non-developable surfaces. All examples evolved from an initial value with a locally non-smooth and complex topological configuration to a smooth result while maintaining high magnetic field accuracy.

## 1. Introduction

MRI is widely used in medical imaging for its high accuracy and due to the fact that it utilizes non-ionizing radiation. One of MRI’s basic components, gradient coils, encode the spatial information of a signal during imaging. This process provides localization information of the MR images. The earliest coils to provide gradient magnetic fields had a simple structure and were developed by Maxwell and Golay, e.g., Maxwell coil pairs and Golay coils [1,2]. Medical imaging has grown in sophistication since its first introduction; similarly, the design method of gradient coils has become more and more complex, which means the number of contours has also increased. One of the most widely used methods through which to determine optimal coil loop contours for a specific current-carrying surface is the stream function method (SFM) [3,4,5].

The SFM transforms the coil design problem into the calculation of the contour lines of a scalar stream function (SF), which is defined on a certain current-carrying surface geometry. The SFM uses the function values related to the SF to represent the current density of the design surface. After discretizing the process, a minimum least squares problem is applied, and the coil structural design problem is transformed into a numerical problem represented by a matrix equation. The SFM was initially only applicable to geometric surfaces such as planes, cylindrical surfaces, and super-elliptical cylindrical surfaces [6,7], which are developable surfaces (a developable surface is a surface that can be flattened onto a plane without stretching or distorting). In recent years, however, with the expansion of MRI applications, traditional integrated body MRI systems no longer meet the demand for high-precision localized MRI imaging in clinical and research settings. Thus, insert gradient coils were developed to generate stronger and faster gradient fields. The gradient slew rate is directly proportional to the inverse of the coil radius’ fifth power [2,8]. Thus, in order to further reduce the coil size and increase spatial efficiency, conformal surfaces are used as the current-carrying surfaces for the gradient coils. In MRI, a conformal surface refers to a surface that has the same shape as the imaging object. These surfaces are typically non-developable. Subsequently, Ren [9] extended the SFM’s application range to non-developable surfaces such as spherical surfaces and human head surfaces by applying external tangential gradient operators and Delaunay meshes.

However, in practical optimization processes, the SFM faces an unavoidable issue. The matrix used for solving the optimization has a large condition number, which makes the problem ill-conditioned. In order to calculate the solution of the ill-conditioned equation group smoothly, a regularization term [10,11] needs to be introduced to the matrix. Although the introduction of the regularization term can improve the ill-conditioned equation group so that it may be solved easily, it also introduces new problems: the selection of the optimal regularization coefficient. A regularization coefficient that is too large reduces the main objective value of the coil optimization function and decrease magnetic field accuracy, but too small of a regularization coefficient results in non-smooth contour lines and generates reverse loops. Due to the non-smoothness of contour lines often occurring only in localized regions of the design domain, we also term this occurrence as “local oscillation of the contour line”. These non-smooth contour lines and reverse loops increase the thermal effects of the coil, and also increase the cost of coil post-processing, which we hope to avoid as much as possible. During the manufacturing process of the coil, an initial configuration with poor smoothness can also lead to an increase in the final magnetic field error. Therefore, balancing magnetic field accuracy and coil smoothness has always been a research point for gradient coil design [12].

The previous solution of this type of problem was to sample enough regularization parameters for optimization, and then obtain the Pareto curve for the optimization main objective versus the regularization term [6]. After gradual iterative calculations, the regularization parameter selected is near the inflection point of the curve. Finally, a smoother result can be obtained within the magnetic field accuracy design range. However, this method is more cumbersome due to its computational cost. The data composition and post-iteration of the L-curve require a great deal of repeated calculations and manual adjustments. Therefore, this article is committed to exploring another method, whereby the idea of smoothing to transform the problem of parameter value regularization into a more direct SF smoothing problem is used. This method obtains a coil structure that meets the design goal and does not have non-smooth contours or reverse loops in a more convenient and direct way. Thus, coil designers can quickly obtain an initial solution that has local oscillations in the preliminary design, and one can then improve these local oscillations via smoothing.

While solving partial differential equations on the surface, the finite element method is widely used to discretize the surface geometry. The finite element method typically considers a single discrete triangle mesh as a plane, ignoring the geometric characteristics of the entire surface. For a design domain of a flat or developable surface, this problem can be ignored. However, in an undevelopable surface design domain, considering discrete triangles as planar surfaces results in loss of curvature and results in other undevelopable characteristics. One of the reasons why the Delaunay mesh is widely used for surface discretization is that it can make the normal vectors of mesh nodes converge to the initial surface, which leads to an external coil design scheme [9]. However, the external method does not consider node curvature information and is heavily dependent on the accuracy of discrete surface normal vectors. It is not suitable for the SF smoothing discussed in this article. Therefore, we introduce the Laplace–Beltrami operator ${∆}_{\Gamma }$, and provide an intrinsic solution to a surface Laplace problem.

To improve the efficiency of coil design, we simplify the complexity of post-processing and reduce the accuracy loss caused by complex post-processing; this is necessary to smooth the SF contour lines. To achieve this goal, this article proposes an algorithm based on intrinsic operators so as to design and smooth SFs for any continuous surface.

## 2. Methods

**Figure 1:**
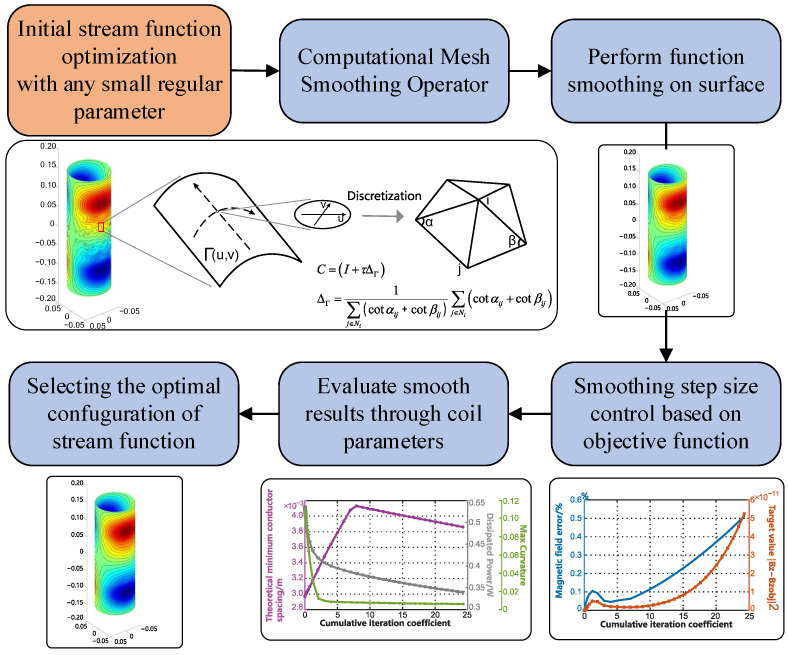
Flowchart describing the various steps of SF smoothing on a surface.

In this section, we develop a general method for smoothing functions on surfaces, which are represented by triangular meshes. The proposed algorithm consists of several steps: optimization of the SF, extraction of the surface mesh information, a smoothing of the operator structuring, a smoothing process and loop parameter calculation, and finally an evaluation of the smoothing effectiveness based on the curvature change in the contour lines of the SF. Figure 1 illustrates the process of these steps.

### 2.1. Previous Work

The SFM is one of the most widely used methods in gradient coil design, and its current-carrying surface is mainly a developable surface. Applying the SFM to undevelopable surfaces via the extrinsic method has also been discussed in detail in Ren’s paper [13]. This work was aimed at coil designs on undevelopable surfaces via the intrinsic method, but it is also compatible with developable surfaces. The SFM on non-developable surfaces is an important prerequisite for the implementation of this work. Therefore, we provide here a brief explanation of the SFM, as well as the issues related to this article.

The SFM simplifies the complex calculations in direct problems by converting the current density vector into a scalar SF. The conversion relationship is represented by the following formula:

(1) $$j=\nabla_{\Gamma }\Psi \times n,$$

where j represents the surface current density; $\nabla_{\Gamma }$ is the tangential gradient operator on the current-carrying surface; Ψ is a scalar stream function that is defined on the surface; and n represents the unit normal vector of the surface.

The core of the SFM is to find a suitable scalar function Ψ, such that that the corresponding surface current density structure can produce a magnetic field that meets design requirements. This can be expressed as an optimization function:

(2) $$min:f={\left\|B_{z}-B_{Target}\right\|}^{2}+\lambda F_{1}.$$

Equation (2) shows a standard optimization problem, where ${\left\|B_{z}-B_{Target}\right\|}^{2}$ is the main objective and $F_{1}$ is a regularization term used to solve ill-posed problems [10,14]. And $B_{z}$ is a function of Ψ, representing the actual magnetic field generated by the coils in the imaging region; $B_{Target}$ is a set of constants representing the target gradient magnetic field within the imaging region; $F_{1}$ represents the auxiliary objective; and λ is the regularization parameter, which is a key factor determining the smoothness of the coil. 

We discretize the current-carrying surface in Equation (2) and choose the coil power consumption as the auxiliary objective. Thus, $B_{z}=B_{ji}\Psi$ and $F_{1}=\frac{1}{2}\;{\left\|C_{Heat}\Psi \right\|}^{2}$, where $B_{ji}$ is the sensitivity matrix of magnetic field $B_{z}$ with respect to Ψ, and $C_{Heat}$ is the power consumption matrix, which uses coil energy consumption as the auxiliary objective. The discrete expression of the above equation is as follows:

(3) $$min:f={\left\|B_{ji}\Psi -B_{Target}\right\|}^{2}+\lambda {\left\|C_{Heat}\Psi \right\|}^{2}.$$

We can obtain the solution of Equation (3) by setting the first-order derivative $\frac{\partial f}{\partial \Psi }$ of the SF to zero. In this step, the choice of the optimal regularization parameter plays a crucial role in determining the weight balance between the two optimization objectives. Solutions obtained with smaller values of λ may achieve lower main objective values, but they may also result in coil structures with a poor smoothness. On the other hand, larger values of λ tend to make the coils smoother but introduce more errors in magnetic field linearity:

(4) $$E_{l}=\frac{\max\left|B_{z}-B_{Target}\right|}{\max\left|B_{Target}\right|}\times 100\%.$$

Therefore, in each instance of SFM, we need to balance the solution by adjusting the value of λ so as to balance magnetic field accuracy and coil power consumption. Thus, a smooth coil configuration can be obtained.

In order to choose an appropriate regularization parameter, it is often necessary to sample it multiple times and obtain an L-curve, which shows the relationship between the main and auxiliary objectives for the different values of the regularization parameter. In this way, we can select the λ value that satisfies the design objective from around the inflection point of the L-curve. Although this method can achieve the purpose of coil optimization design, it results in iteration calculations that uses multiples, which greatly increases the time and labor cost of the coil design. In contrast, smoothing methods can make the SF configuration evolve towards smoothness through linear calculations. Using this method, it only takes a short time to evolve a poorly smooth initial SF configuration to a smooth SF configuration.

### 2.2. Smooth Function on a Surface

The smoothness of the SF contour has always been an unavoidable research topic in coil design. The Tikhonov regularization method is a solution to this topic [10,14,15]. Another method for smoothing SF contours is to use a smoothing operator, where the simplest form is the filtering method, which averages the stream function values of each node by applying certain weights [16,17]. The weights are usually assigned according to the distance information between the node and its neighbors in a well-defined neighborhood. This method changes the interpolation of the equivalent points within the unit by filtering the SF values of the nodes; thus, the SF contour within the design surface tends to be smooth. Before conducting the work in this paper, we attempted to use distance weighting for function filtering on non-developable surfaces, but we were unable to obtain a smooth contour solution. This is because the filtering matrix composed of distance weighting only includes the relative position information of the points on the surface and ignores the curvature degree of the surface itself.

Therefore, a more general smoothing operator is preferred that could include the geometric information of the current-carrying surface, such that the smoothing of the SF within the surface can be conducted inside only a manifold. An important property of SF is that it is embedded in the current-carrying surface. In the process of smoothing evolution, the smoothing operator needs to preserve the embedding property and use the geometric characteristics of the current-carrying surface to smooth the SF. In this way, the structure of the SF contour lines along the surface scale can be simplified [18].

We let Γ be a two-dimensional manifold embedded in $IR^{3}$; $\left(u,v\right)\in \Gamma$ be the coordinates in a two-dimensional manifold; and let $\Psi \left(u,v\right)$ be the SF defined on the surface Γ [19]. We then need to evolve the implicit function $\Psi \left(u,v\right)$ such that its contour line, $\Psi \left(u,v\right)=\Psi_{i}$, evolves toward a geodesic curve, where $\Psi_{i}\in \Psi \left(u_{i},v_{i}\right)$ is the SF value at a certain point $\left(u_{i},v_{i}\right)$ on the surface. Such problems have been described when using the heat diffusion equation that balances changes in the concentration in space [20]:

(5) $$\frac{\partial \Psi }{\partial t}=∆\Psi ,$$

where t represents the time of diffusion. Essentially, this involves using a filtering method with a second-order PDE. In the non-developable surface designed in this work, we need to extend the Laplace operator onto two-dimensional manifolds, where the Laplacian operator ∆ needs to be replaced by the Laplace–Beltrami operator ${∆}_{\Gamma }$ [21]. Then, the diffusion equation for functions on the surface Γ is

(6) $$\frac{\partial \Psi }{\partial t}-{∆}_{\Gamma }\Psi =0.$$

Equation (6) needs to be discretized in the time domain as follows:

(7) $$\frac{\Psi^{n+1}-\Psi^{n}}{\tau }={∆}_{\Gamma }\Psi^{n},$$

where τ represents the time step. After a simple derivation, the following form can be obtained:

(8) $$\Psi^{n+1}=\left(I+\tau {∆}_{\Gamma }\right)\Psi^{n}.$$

Thus, we obtain the smoothing operator:

(9) $$C=\left(I+\tau {∆}_{\Gamma }\right).$$

**Figure 2:**
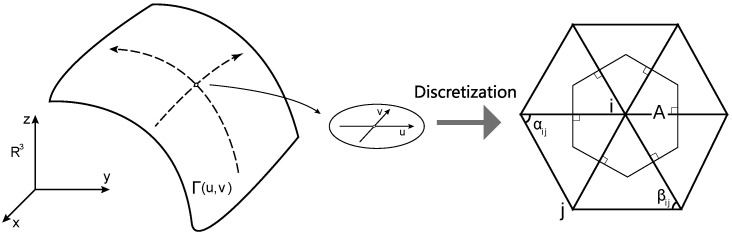
The L–B operator discrete explanatory diagram.

This means that we only need to solve a linear equation to achieve the smoothing of the surface functions. Here, I is the identity matrix, τ is the time step, and $\Psi^{n}$ denotes the function value on the two-dimensional manifold Γ at time t=nτ in the time domain. The Laplace–Beltrami operator in the discretized surface representation can be calculated using the following equation [21]:

(10) $${∆}_{\Gamma }=\frac{1}{2A}\sum_{j\in N_{i}}\left(\cot\alpha_{ij}+\cot\beta_{ij}\right),$$

where$\;\alpha_{ij}$ and $\beta_{ij}$ are the angles of the triangle formed by the vertices of the mesh edge ij and A is the actual neighborhood area under the control of node i, as shown in Figure 2. For ease of illustration, a planar triangular mesh is shown in Figure 2. In practice, the L–B operator is usually applied in two-dimensional manifolds, that is, $\sum_{j}\left(\alpha_{ij}+\beta_{ij}\right)\geq 2\pi$ for a convex surface.

To speed up the smoothing process, this work normalizes the operator by using Desbrun’s method [17], which allows for the use of larger time steps in explicit integration methods, as shown in Equation (10):

(11) $${\left({∆}_{\Gamma }\Psi \right)}_{normalized}=\frac{\sum_{j\in N_{i}}\left(\cot\alpha_{ij}+\cot\beta_{ij}\right)\left(\Psi_{i}-\Psi_{j}\right)}{\sum_{j\in N_{i}}\left(\cot\alpha_{ij}+\cot\beta_{ij}\right)}.$$

### 2.3. Smoothing Coefficients Based on the Objective Function Control

In the previous section, we normalized the time step for each iteration. However, in practical applications, there is often an abrupt decrease in the accuracy of the magnetic field during the initial stages of the smoothing iteration. Maintaining a constant smoothing step size can easily lose the documented changes occurring during the iteration process, resulting in a rapid loss of accuracy in the coil’s magnetic field. Therefore, the main objective function of the coil design process in this work is introduced to assist in the selection of the smoothing step size:

(12) $$F_{0}^{n}={\left\|B_{z}^{n}-B_{Target}\right\|}^{2},$$

where $F_{0}^{n}$ represents the main objective value for the nth iteration step. Before each iteration in the program, an estimate of the smoothed main objective value $\bar{F_{0}^{n+1}}\;$ is calculated. Then, the program determines the value of the iteration step τ based on the size of the objective value in reverse.

(13) $$\tau =\left\{\begin{array}{cc} \max\left(0.01,\frac{F_{0}^{n}}{\bar{F_{0}^{n+1}}}\right) & \bar{F_{0}^{n+1}}\geq F_{0}^{n}\\ 1 & \bar{F_{0}^{n+1}}<F_{0}^{n} \end{array}\right..$$

That is, the program adjusts the size of the time step τ for each iteration in real time during the smoothing process. If the increase in the objective function is too large, a smaller step size coefficient (no less than 0.01) is assigned. If the increase in the objective function is relatively small, a larger step size coefficient (no greater than 1) is assigned to accelerate the iteration. Certainly, the objective function may be decreased during the smoothing process. In this case, we assign the maximum step size coefficient, which is 1.

### 2.4. Controlling the Spacing between Contour Lines Based on the Tangential Gradient

In most cases of gradient coil design and manufacturing, we want to maintain a large number of wire turns to reduce the current value of the wires and maintain the stability of the magnetic field formed by the coil. However, in the case of a high number of wire turns, some areas will have wire layouts that are too dense. This can easily cause an increase in the inductance of the coil. Due to the frequent switching of gradient coils during the operation of MRI equipment, coils generating the gradient magnetic fields should have low inductance [22,23]. The method of reducing the density of the coil is an empirical way to reduce inductance. As mentioned earlier, the contour lines in the SFM are the coil structure. Therefore, reducing the sampling interval of the contour lines can easily reduce the density of the coil. However, this will also decrease the coil density in already sparse areas. Of course, we do not want to excessively sacrifice the accuracy and stability of the magnetic field to reduce the inductance. Fortunately, during the diffusion process of the function, the coil layout naturally evolves toward uniformity. All we need to do is monitor the changes in wire spacing in real time during the design process. Thus, given the number of coil turns, we can select a smooth result that satisfies the design conditions while maximizing the wire spacing between the wires. This makes it an important indicator for assisting in the selection of a smooth result. In this work, the gradient operator ∇ is used to perform first-order gradient calculations on the SF. The theoretical minimum wire spacing that can be achieved under the current result can be estimated by combining the numerical value of the SF with the number of discrete wires as follows:

(14) $$d_{min}=\frac{\left(\Psi_{max}-\Psi_{min}\right)/N_{Wires}}{{\left|\nabla_{\Gamma }\Psi \right|}_{max}},$$

where the $N_{Wires}$ is the number of discrete coil turns and $\nabla_{\Gamma }\Psi$ is the tangential gradient vector of the SF along the surface. By using the tangential gradient operator, we can monitor the changes in wire spacing during the smoothing process of the SF on any surface. This allows us obtention of the optimal number of discrete coil turns that meet the design goals.

### 2.5. Curvature Changes in Implicit Contour Expression

The goal of this work is to smooth the SF contour lines. Thus, this section describes a quantitative index for smoothing. The smoothness of a curve in space at any point can be expressed by its curvature. A higher curvature at a point indicates that the curve is more bent at that position, while a lower curvature at a point indicates a smoother curve. This section calculates the implicit curvature value of the contour at each node on the current-carrying surface. Thus, the maximum value of the implicit curvature is used as the index for the level of function smoothness.

In the SFM, the contour lines of the SF at any point inside the coil design surface Γ can be implicitly expressed by the function $\Psi -\Psi_{i}=0$, where $\Psi_{i}$ can take on any value of the SF within the design domain and where $\Psi_{i}\in \left[\Psi_{min},\Psi_{max}\right]$. Thus, the curvature value of the curve can be calculated by using a second-order differential of its implicit function expression:

(15) $$\kappa ={∆}_{\Gamma }\Psi .$$

During the smoothing process, the maximum curvature value max⁡(κ) on the current-carrying design domain gradually decreases and converges. Based on this, the convergence condition for smoothing can be set to

(16) $$\frac{{\max\left(\kappa \right)}_{i}-{\max\left(\kappa \right)}_{i-1}}{{\max\left(\kappa \right)}_{i}-{\max\left(\kappa \right)}_{1}}<1\%,\;$$

where $\max{\left(\kappa \right)}_{i}$ represents the maximum curvature value of the SF contour configuration in the i-th iteration.

## 3. Numerical Example

This section applies the proposed method to a developable cylindrical surface and an undevelopable human head surface. Furthermore, in both planar and curved structures, smooth SF contour lines that meet design accuracy requirements are generated. Depending on the complexity of the smoothing model, the program takes from several seconds to dozens of seconds to run iteratively until convergence on an Intel(R) Core(TM) i5-9300H 2.4 GHz quad-core 8-thread CPU. We only need to select the optimal smoothing configuration that meets the design goal based on the physical parameters of the coil during the smoothing process and the variation in the smoothing convergence curve. Considering that the iteration coefficients for each step of the iteration process may differ for different coil models, this work develops a unified iterative variable that ultimately sums all the smoothing coefficients up to the current iteration step. This process represents the timeline of the iteration process, e.g.,

(17) $$coe=\sum_{i=1}^{n}\tau_{i},$$

where coe is the iteration degree variable, *n* is the current iteration step, and $\tau_{i}$ is the time step coefficient for the ith iteration. Thus, we separately present the smoothing process of the SF for undevelopable and developable surfaces. We also demonstrate the changes in various parameters of the coil during the process, including magnetic field accuracy, objective function variation, energy consumption, coil length, minimum wire spacing, maximum curvature of contour lines, etc.

The resulting SF smoothing process is described below.

### 3.1. Undevelopable Human Head Surface Gradient Coil

**Figure 3:**
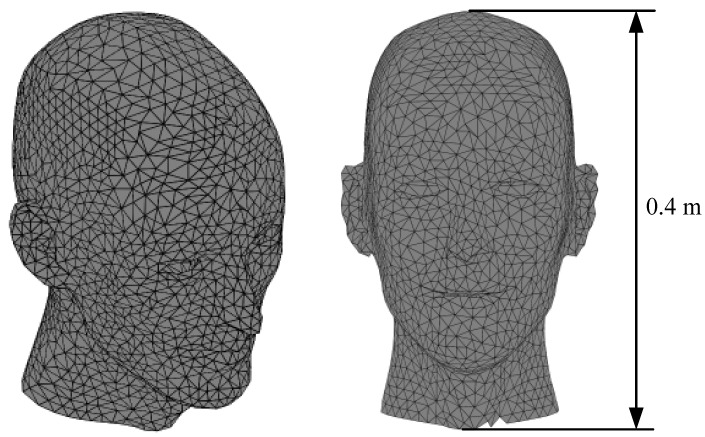
Schematic diagram of the dimensions of the curved surface of the human head.

**Figure 4:**
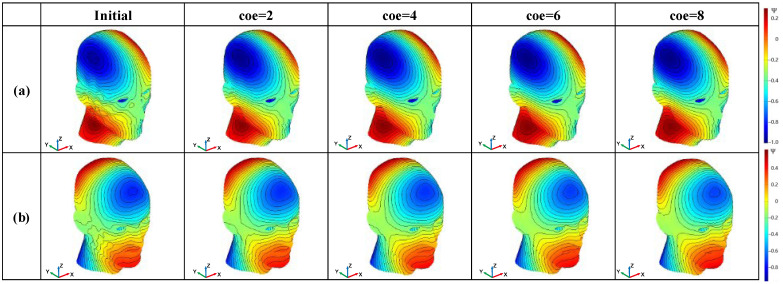
Configuration variation in the SF on the surface of the human head: (**a**) gradient coil in the x-direction of the human head surface; (**b**) gradient coil in the y-direction of the human head surface.

Figure 3 shows the grid and current surface dimensions of the human head surface. This is an undevelopable surface with a height of 0.4 m. The imaging region is a sphere with a diameter of 0.1 m. The target magnetic field gradient is 10 mT/m. Figure 4 shows the smoothing process of the SF configuration for the gradient coil on the undevelopable human head surface. The leftmost column in Figure 4 shows the initial image of the flow function structure, and the process of the SF evolution during the smoothing iteration is shown from left to right.

#### 3.1.1. X-Gradient Coil on an Undevelopable Surface

**Figure 5:**
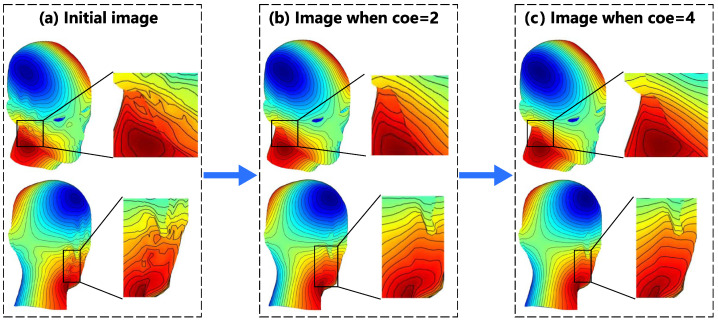
The SF evolution of the gradient coil in the x-direction from initial image to coe=4: (**a**) exhibition of the oscillation regions in the initial configuration of SF; (**b**) demonstration of the smoothing effect when coe=2 on the oscillation regions; and (**c**) demonstration of the smoothing effect when coe=4 on the oscillation regions.

Figure 4a refers to the x-gradient coil on the undevelopable human head surface. For the SF model in Case (a), the smoothing operator shows good results. Figure 5 shows a clearer demonstration of the evolution from the initial image to coe=4. According to Figure 5a, the coil oscillation is mainly concentrated in the facial area connecting to the ears and neck, as well as around the eyes. However, at coe=2 in the smoothing process, the contour lines of the SF in these areas reach basic smoothness, and the smoothing requires 27 steps. After that, the program step size increases, and the smoothing speed also increases. A sufficiently smooth SF configuration is obtained at coe=4.

**Figure 6:**
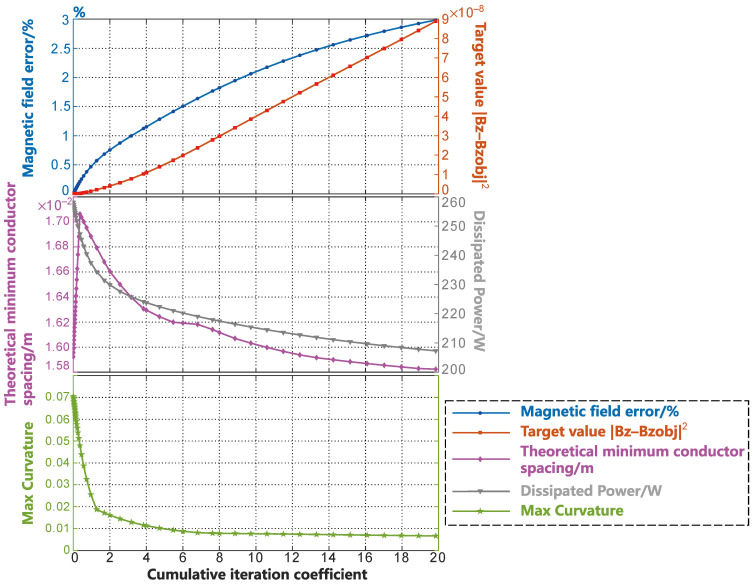
Variation in the parameters during the smoothing process of the gradient coil in the x-direction within the head form surface.

**Figure 7:**
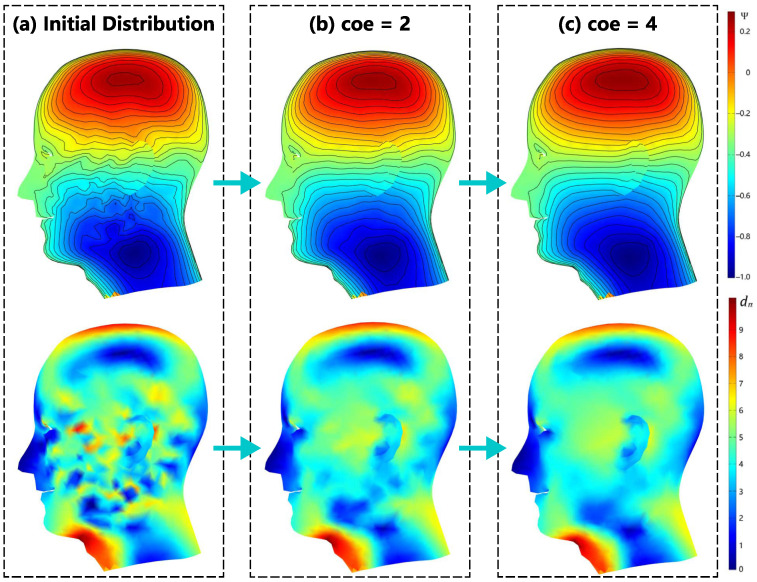
The SF contour lines and corresponding wire spacing distribution from the initial value to coe=4: (**a**) the initial SF contour lines configuration (**top**) and wire spacing distribution (**bottom**); (**b**) the SF contour lines configuration (**top**) and wire spacing distribution (**bottom**) when coe=2; and (**c**) the SF contour lines configuration (**top**) and wire spacing distribution (**bottom**) when coe=4.

Figure 6 shows the changes in the various parameters of the coil during the smoothing process. In Figure 6, the blue curve shown by the circular markers at the top represents the percentage of magnetic field error, while the red curve shown by the square markers represents the optimization objective value for the coil. The increase in the magnetic field error at coe=4 is less than 1.5%, and the corresponding increase in the optimization objective value is within $1\times 10^{-8}$. In the middle of Figure 6, the purple curve with diamond markers represents the change in the minimum wire spacing, while the gray curve with inverted triangle markers represents the change in the energy consumption of the coil during the smoothing process. The change in the wire spacing curve occurs due to the narrow width of the x-direction of the designed surface of the human head, which is different from the y-gradient coil. In this section, the changes in the distribution of wire spacing are further elucidated through Figure 7. In the wire spacing distribution shown in Figure 7, the darker the red color, the denser the wire distribution. Thus, during the initial stage of the smoothing process, the minimum wire spacing is always located above the head and in front of the neck. As the SF contour lines gradually smooth out, the areas of dense wire spacing caused by the oscillations disappear. As they are affected by the SF diffusion, the SF contour lines in front of the neck become denser. As a result, the purple curve, which represents the minimum wire spacing in Figure 6, decreases. After a certain period of time in the smoothing process, the SF contour lines start to diffuse toward the sparse regions, resulting in an increase in the wire spacing above the head and in front of the neck. This is reflected in Figure 6 by the rise in the purple curve after coe=7.

The energy consumption during the smoothing process also rapidly decrease as the coil oscillation disappears during the smoothing process. The change in the maximum curvature of the SF contour lines during the smoothing process is shown by the green curve with star markers in the bottom of Figure 6. The inflection point of the maximum curvature curve appears in the coe=2−4 stages. The convergence condition described by Equation (15) is satisfied at coe=4. Considering the various curve parameters shown in Figure 6, the SF configuration obtained at coe=4 is undoubtedly the best in this example. At this stage, the coil efficiency $\left(G_{x}/\left|r_{ROI}\times I\right|\right)$ [2] is $\frac{0.72073}{a^{2}}\;mT\cdot m^{-1}\cdot A^{-1}$. Here, $G_{x}=dB_{z}/dx$ is the gradient value of the magnetic field in the x-direction; $r_{ROI}$ denotes the radius of the imaging region; and a represents the average radius of the coil design surface.

The quality of the mesh partition for the undevelopable surfaces affects the calculation accuracy of the L–B operator. A poor mesh partitioning introduces larger normal surface errors, which accelerates the loss in the magnetic field accuracy during the smoothing process. Therefore, for the non-developable surface in the example, we use the Delaunay triangulation method [24,25]. Thus, the error tolerance relative to the target magnetic field is controlled within 5% to meet the design requirements of the gradient coil.

#### 3.1.2. Y-Gradient Coil on Undevelopable Surface

**Figure 8:**
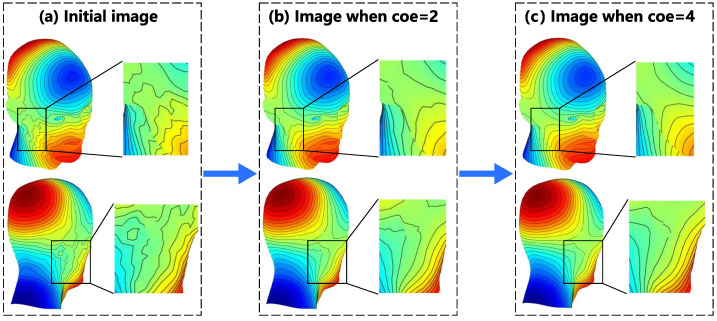
The SF evolution of the gradient coil in the y-direction from the initial image to coe=4: (**a**) exhibition of oscillation regions in the initial configuration of SF; (**b**) demonstration of the smoothing effect when coe=2 in the oscillation regions; and (**c**) demonstration of the smoothing effect when coe=4 in the oscillation regions.

Figure 4b shows the y-gradient coil configuration and its smooth iteration on the same surface as in example (a). Due to the poor symmetry of the current-carrying surface, rotations of the target magnetic field along the non-symmetric axes can cause significant changes in the SF configuration. This is also the reason why we chose it as a separate example. From the initial image shown in Figure 8a, the oscillations in the initial SF configuration mainly occur on the facial surface and the lateral side. These oscillations need to be eliminated during the coil design process.

**Figure 9:**
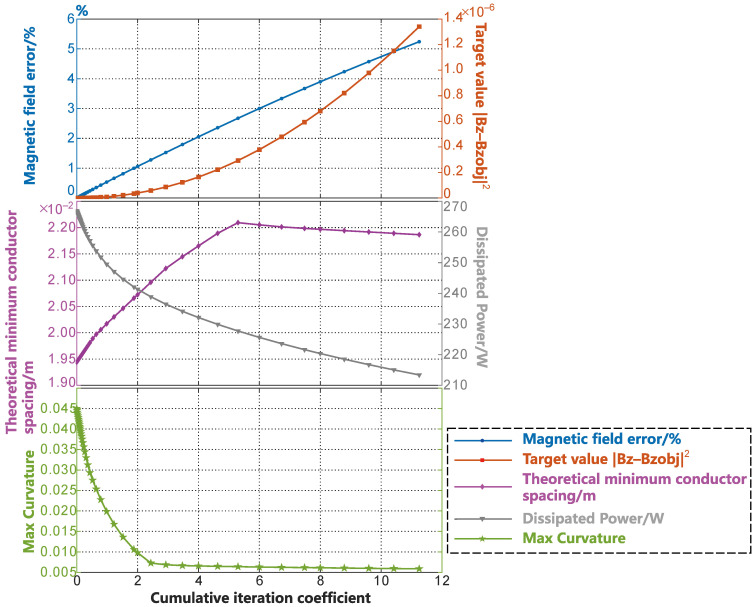
Variation in the parameters during the smoothing process of the gradient coil in the y-direction within the head form surface.

**Figure 10:**
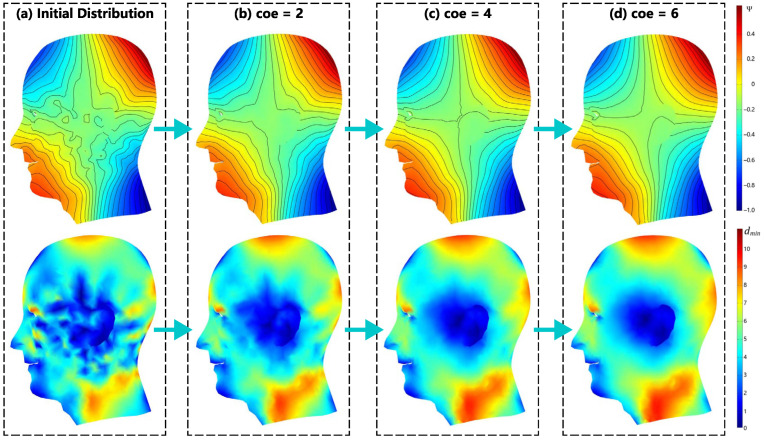
The SF contour lines and corresponding wire spacing distribution from the initial value to coe=6: (**a**) the initial SF contour lines configuration (**top**) and wire spacing distribution (**bottom**); (**b**) the SF contour lines configuration (**top**) and wire spacing distribution (**bottom**) when coe=2; (**c**) the SF contour lines configuration (**top**) and wire spacing distribution (**bottom**) when coe=4; and (**d**) the SF contour lines configuration (**top**) and wire spacing distribution (**bottom**) when coe=6.

From the smoothing process in Figure 8b, when the time step reaches coe=2, the coil oscillation and independent loop on the designed surface disappears, and the coil is essentially smooth. At this point, the iteration progresses to 30 steps. In Figure 8c, when the process reaches coe=4, the smoothness of the coil essentially meets the requirements for post-processing. At this point, the error loss generated by the coil-generated magnetic field relative to the target magnetic field is less than 2%, as shown by the blue curve with circular markers in Figure 9. The image in the middle of Figure 9 shows the variation in the theoretical minimum wire spacing and energy consumption of the coil, which is indicated by the purple and gray curves, respectively. Figure 10 provides a detailed explanation of the variation in the minimum wire spacing curve. Unlike the case in the x-direction, the wire spacing distribution in this example is more scattered. As the smoothing process progresses, the SF contour lines for the top of the head, back of the head, sides of the neck, and upper eyelids gradually becomes more dispersed. This leads to a gradual increase in the purple curve, as shown in Figure 9. The energy consumption of the coil decreases continuously with the smoothing process, which is also the desired result for the designers. In the bottom of Figure 10, the green curve, which represents the maximum curvature of the SF contour lines, gradually decreases and meets the convergence condition at coe=5.32, indicating that the curve is smoothed to a certain extent. At that point, the coil efficiency is $\frac{0.5382}{a^{2}}\;mT\cdot m^{-1}\cdot A^{-1}$.

### 3.2. Cylindrical Developable Surface Gradient Coil

**Figure 11:**
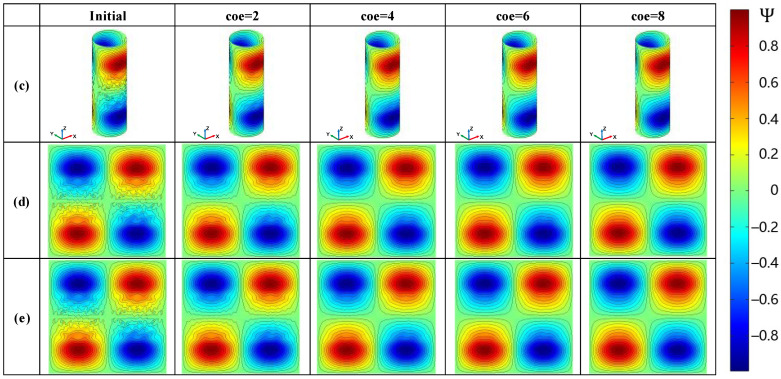
Configuration of the variation in SF in the expandable cylindrical surface: (c) the design and smoothing of gradient coils for an unfolded cylindrical surface; (d) the busbar expansion diagram of Example (c); and (e) the design and smoothing of the gradient coils for a cylindrical surface.

To demonstrate the compatibility of the proposed method on planar meshes, this section presents the SF configuration for two types of radial gradient coils, i.e., folded and unfolded, on the same size cylindrical design surface along with their corresponding smoothing processes. Both coils were initialized with high accuracy and low smoothness through the same Tikhonov regularization minimization. Subsequent iterations of the smoothing were based on this initial value. The coil cylinder had a height of 0.3 m and a diameter of 0.1 m. To maintain the compactness of the coil, the imaging area was a sphere with a uniform diameter of 0.08 m. The magnetic field gradient value of the coil was designed to be 10 mT/m. Figure 11 shows the initial value of the SF configuration for the gradient coil and the corresponding iterative smoothing process. To compare the cylindrical Case (c) more intuitively with the planar Case (e), the results of the cylindrical case were unfolded along the axis and shown in Figure 11(d). It is necessary to explain here that although Case (c) and Case (e) had the same physical and dimensional parameters for the design surface and imaging area, as well as the same regularization coefficient, due to the difference in the calculation of the sensitivity matrix $\left[\partial B_{z}/\partial \Psi \right]$ on the curved surface and the planar grid, it was not possible to obtain the same initial value. This section tries to control the physical parameters as much as possible to reduce this difference.

#### 3.2.1. Cylindrical Gradient Coil

**Figure 12:**
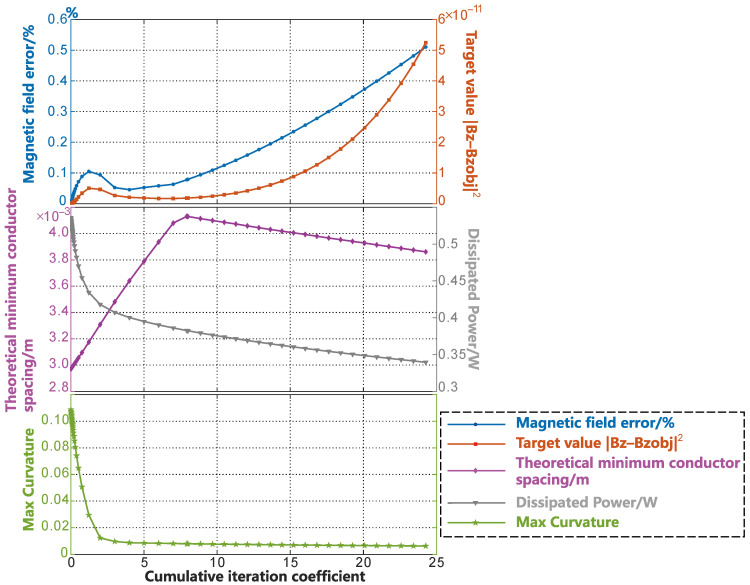
Variations in the various parameters in the smoothing process of SFs on a cylindrical surface.

**Figure 13:**
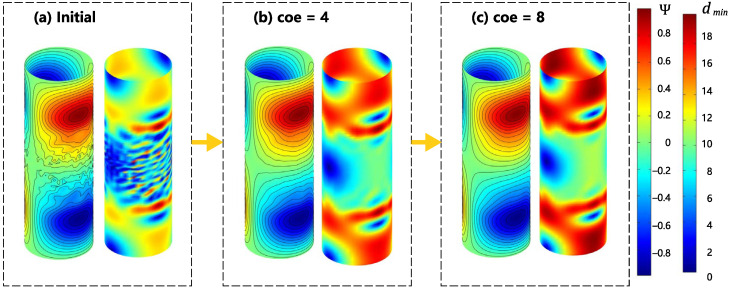
The evolution of the SF configuration and the corresponding conductor spacing from the initial value to coe=8.

Figure 11 (c) shows the smoothing process of the radial gradient coil on the cylindrical surface before unfolding. The smoothing in Case (c) was conducted entirely within the unrolled cylinder surface. On the cylindrical surface, there were many oscillations and independent coil loops in the middle part of the contour lines of the coil due to the selection of the low regularization coefficient. We can see that during the smoothing process, the independent coil loops gradually disappeared, and the SF contour lines changed from oscillating to smooth, then finally become completely smooth. The change in the coil parameter during the smoothing process is shown in Figure 12, and the error of the magnetic field accuracy compared to the objective value was within 0.6%. The optimized objective value gradually increased during the smoothing process, but it can be maintained within the order of $10^{-10}$. The minimum wire spacing gradually increased in the initial stage of smoothing. To better illustrate the variation in the theoretical minimum wire spacing, which is represented by the purple curve in Figure 12, we plotted Figure 13. In each subplot of Figure 13, the left side corresponds to the SF configuration of the respective optimization stage. The images on the right side represent the wire spacing distribution that corresponds to the SF configuration. In these images, the darker the red color, the denser the wire distribution in that area. In the initial SF configuration shown by Figure 13a, the position of the minimum spacing between the wires appeared between two sets of coils along the axis of the coil. As the coil further evolved toward uniformity, the distribution of the wire spacing gradually became uniform. The minimum spacing position shifted toward the outer end of the radial coil. This is reflected in the purple curve of Figure 12 as a slow reduction in the wire spacing after the inflection point. The energy consumption of the coil decreased rapidly as the small independent coil loops disappeared and the boundary was gradually smoothed. After it decreased to 0.4 W, the rate of decrease gradually slowed down. The maximum curvature change in the SF contour lines, as shown by the green curve with star markers in Figure 12, indicated that the contour lines were rapidly smoothed in stages of coe=0−3, and the convergence condition was met when coe=4. The smoothing of the entire coil reached a certain degree.

To demonstrate the convergence of the smoothing program and the changes in the parameters of the coil, a sufficient number of iterations were run in this article. However, in the actual coil design, we only needed to select a good result based on the convergence condition and comprehensive consideration of the changes in various parameters during the smoothing process. Usually, this process only takes a few seconds to complete. In this case, as shown in Figure 12, to achieve a larger wire spacing, the coil structure with a coe=6 could be set as the optimal result for the smoothing. This came at the cost of a relative loss of accuracy in the target magnetic field of less than 0.1%, and the value of the coil was then $\frac{1.8687}{a^{2}}\;mT\cdot m^{-1}\cdot A^{-1}$. In this example, the design surface radius was set as a=0.05 m.

#### 3.2.2. Cylindrical Unfolded Gradient Coil

**Figure 14:**
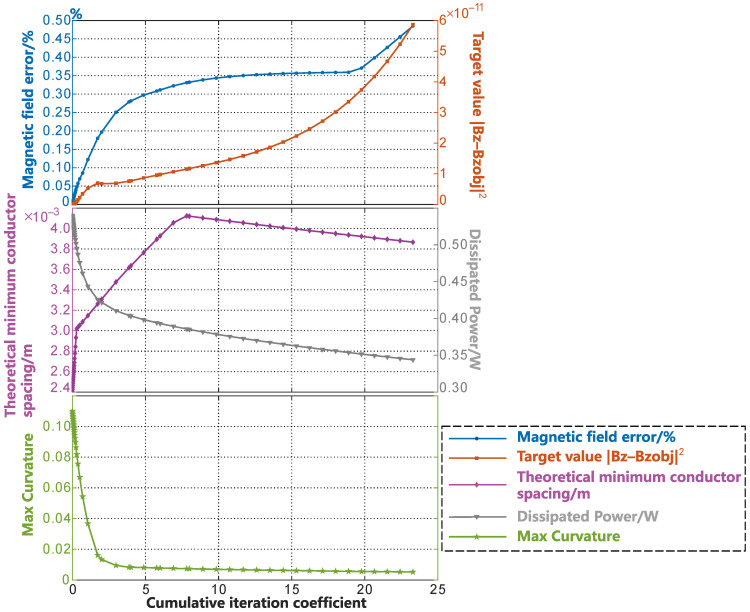
Variations in the various parameters in the smoothing process of SFs on an unfolded cylindrical surface.

Figure 11 (e) shows the smoothing of the gradient coil on the unfolded plane of the cylindrical coil. Example (e) has the same number of grid cells and division method as that in Example (c), as well as the same regularization coefficients and SF structure. The difference is that the smoothing process of Example (e) is completely carried out on the unfolded plane. The variation in the coil parameters during the smoothing process is shown in Figure 14.

By comparing Example (d) and (e) in Figure 11, the SF on the curved surface and the unfolded plane underwent a similar smoothing evolution process and degree. However, the coil parameters in Figure 14 reveal more details regarding the smoothing process. The same initial value of the gradient coil showed a similar but not identical smoothing process due to the different shapes of the curved surfaces. The latter stages of the SF evolution process on the unfolded plane even had a lower error loss. The curve of the objective function, as shown by the red curve with square markers in Figure 14, maintained a similar shape and the same order of magnitude as in Example (c). The minimum wire spacing is shown by the purple curve with diamond markers in Figure 14. Although Example (e) had a lower initial value and a larger increase rate in the initial stage of smoothing due to the slight difference in the initial value, it had a similar turning point and the same geometric meaning as Example (c) in general. The changes in coil power consumption and the maximum curvature value of the contour lines were almost identical in both examples. When choosing the smoothing parameter coe=6 for both examples, the accuracy loss relative to the target magnetic field was less than 0.1%. In this example, the coil efficiency was $\frac{1.8638}{a^{2}}mT\cdot m^{-1}\cdot A^{-1}$, which was almost the same as in Example (c).

When comparing the two sets of examples on the cylindrical surface and its unfolded plane—although the smoothing operator in this paper had a different evolution process when applied to the surface and the unfolded plane due to the curvature of the design surface grid—the smoothing operator undoubtedly produced the same smoothing performance on the unfolded plane.

## 4. Discussion and Conclusions

The work presented in this paper proposes an alternative method for constructing smooth MRI gradient coils when using Tikhonov regularization. In contrast to the most used L-curve method, the entire smoothing process for our proposed algorithm takes only a few seconds to tens of seconds when utilizing the given initial value of the SF.

This method is successfully applied to gradient coil design on complex conformal surfaces, and it is shown to be compatible with classical cylindrical surfaces and their unfolded planes. This method constructs a diffusion equation of the implicit function on the surface, ensuring function smoothness without being restricted by the shape of the surface. The method can be applied to most $C^{0}$ smooth discrete design surfaces, thus avoiding the problem of having to parameterize non-developable design surfaces, which will be greatly beneficial for the development of conformal gradient coils.

## Data Availability

The data are contained within the article.
